# Role of enzalutamide in primary and recurrent non-metastatic hormone sensitive prostate cancer: a systematic review of prospective clinical trials

**DOI:** 10.1038/s41391-024-00829-9

**Published:** 2024-04-08

**Authors:** Mohamed Shelan, Vérane Achard, Felix Appiagyei, Lucas Mose, Thomas Zilli, Christian D. Fankhauser, Constantinos Zamboglou, Osama Mohamad, Daniel M. Aebersold, Richard Cathomas

**Affiliations:** 1 Department of Radiation Oncology, Inselspital Bern, University of Bern, Bern, Switzerland; 2 Faculty of Medicine, University of Geneva, Geneva, Switzerland; 3 Department of Radiation Oncology, HFR Fribourg, Villars-sur-Glâne, Switzerland; 4 Department of Radiation Oncology, Oncology Institute of Southern Switzerland, EOC, Bellinzona, Switzerland; 5 Faculty of Biomedical Sciences, Università della Svizzera italiana, Lugano, Switzerland; 6 Department of Urology, Luzerner Kantonsspital, University of Lucerne, Lucerne, Switzerland; 7 Department of Radiation Oncology, Medical Center – University of Freiburg, Faculty of Medicine, University of Freiburg, Freiburg, Germany; 8 German Oncology Center, University Hospital of the European University, Limassol, Cyprus; 9 Department of Genito-urinary Radiation Oncology, MD Anderson Cancer Center, Houston, TX USA; 10 Department of Oncology/Hematology, Kantonsspital Graubünden, Chur, Switzerland

**Keywords:** Urogenital diseases, Prostate cancer

## Abstract

**Introduction:**

Enzalutamide, a second-generation androgen receptor inhibitor, is indicated for the treatment of metastatic disease, as well as in the treatment of non-metastatic castration resistant prostate cancer (PCa). This systematic review aims to determine outcomes and toxicity in patients with non-metastatic castration sensitive prostate cancer (nmCSPC) treated with enzalutamide in the primary or salvage settings.

**Method:**

We performed a systematic review focusing on the role of Enzalutamide in the treatment of nmCSPC, using the PubMed/Medline database. Articles focusing on androgen receptor inhibitors in nmCSPC were included, while articles discussing exclusively metastatic or castration-resistant PCa were excluded.

**Results:**

The initial search retrieved 401 articles, of which 15 underwent a thorough assessment for relevance. Ultimately, 12 studies with pertinent outcomes were meticulously examined. Among these, seven studies were dedicated to the investigation of enzalutamide in the primary setting, while the remaining five publications specifically addressed its use in salvage settings. Regardless of the treatment setting, our data revealed two distinct therapeutic strategies. The first advocates for the substitution of enzalutamide for androgen deprivation therapy (ADT), based on the premise of achieving equivalent, if not superior, oncological outcomes while minimizing treatment-related toxicity. The second, adopting a more conventional approach, entails augmenting the effectiveness of ADT by incorporating enzalutamide.

**Conclusion:**

Enzalutamide has considerable potential as a therapeutic strategy for nmCSPC, either used alone or in combination with ADT in the primary or in the salvage settings. The use of enzalutamide instead of ADT is an appealing strategy. However, more trials will be required to further understand the efficacy and side-effect profile of enzalutamide monotherapy.

## Introduction

Prostate cancer (PCa) is the third cause of cancer deaths in men from the European Union with 78,800 deaths in 2020. The 2020 PCa mortality rate was 10.0/100,000, declining by 7.1% since 2015 [1]. These favorable trends reflect improvements in treatment strategies. Androgen deprivation therapy (ADT) has been a cornerstone of PCa treatment for several decades. More recently, androgen receptor pathway inhibitors (ARpI), including androgen receptors targeted agents (ARTA) such as Enzalutamide, Apalutamide, and Darolutamide, and steroidogenesis inhibitors such as Abiraterone, have profoundly impacted the management of advanced prostate cancer. They have been shown to delay progression, increase overall survival (OS), and improved quality of life in patients with metastatic castration sensitive PCa (mCSPC) [2], metastatic (mCRPC) [3], or non-metastatic castration resistant PCa (nmCRPC) [4]. Combined with ADT, they have become standard of care in these indications.

After the AFFIRM study results, enzalutamide was initially approved by the United States Food and Drug Administration (U.S. FDA) in 2012 for patients with mCRPC who had previously received docetaxel treatment [5]. Enzalutamide’s indication for treatment was expanded to include mCRPC patients who had never received chemotherapy in 2014 [6]. Enzalutamide was licensed by the U.S. FDA in 2018 for use in patients with nmCRPC as a result of the promising findings of the PROSPER trial [7, 8]. Following the findings of the ARCHES trial, the FDA approved enzalutamide for patients with mCSPC in December 2019 [9]. The benefit of enzalutamide in the mCSPC setting was subsequently confirmed in the ENZAMET trial [10].

The next generation of trials is exploring the benefit of ARpI in non-metastatic PCa, especially in high-risk localized PCa in combination with localized treatment and in patients with biochemical recurrence after local treatment, alone or in combination with androgen suppression or other targeted therapies. This systematic review aims to provide an overview of the available evidence for enzalutamide in the management of non-metastatic hormone sensitive PCa (nmCSPC).

## Methods

This study was conducted in accordance with the Preferred Reporting Items for Systematic Review and meta-analyses (PRISMA) statement [11]. The review protocol was registered in the International Prospective Register of Systematic Reviews (PROSPERO ID: CRD42023402738).

### Study selection

For the bibliographic search, the electronic database Pubmed was used with no time restriction. The final search equation was (prostate cancer[Title/Abstract]) AND ((hormone naive[Title/Abstract] OR hormone sensitive[Title/Abstract] OR castration sensitive[Title/Abstract] OR localized[Title/Abstract] OR non metastatic[Title/Abstract] OR low risk[Title/Abstract] OR intermediate risk[Title/Abstract] OR high risk[Title/Abstract] OR salvage[Title/Abstract] OR recurrent[Title/Abstract] OR relapse[Title/Abstract] OR recurrence[Title/Abstract] OR rising psa[Title/Abstract] OR rising prostate specific antigen[Title/Abstract])) AND ((enzalutamide[Title/Abstract] OR xtandi[Title/Abstract] OR mdv3100[Title/Abstract])) NOT (review[Publication Type]).

Publications were included for review if they were prospective trials exploring outcomes and toxicity of hormone sensitive PCa patients with no evidence of distant metastases treated with enzalutamide. Publications were excluded if they were not written in English; exclusively related to castration resistant PCa; exclusively related to metastatic PCa; or were published as editorials, commentaries, letters, case reports, or trial protocols. If multiple publications were identified describing similar results from the same data set, only the most recent publication was retrieved.

Two authors performed electronic searches of the online MEDLINE database in August 2023. The titles and abstracts were assessed by the same two authors and all potentially relevant articles were identified for full-text review. The final selection from the set of full-text articles was independently performed by two authors with disagreements resolved via discussion with two senior authors.

### Data extraction

Information on each study characteristic was extracted by two authors (V.A and F.A) and included the article information (e.g., first author, publication year), population demographics, study methodology, and key findings. Oncological outcomes as well as enzalutamide-related toxicity were summarized.

### Analysis of results

Studies were analyzed using a narrative synthesis approach. A meta-analysis of OS rates and toxicity was not performed because of the variation in study populations and intervention types in the studies.

## Evidence synthesis

**Fig. 1:**
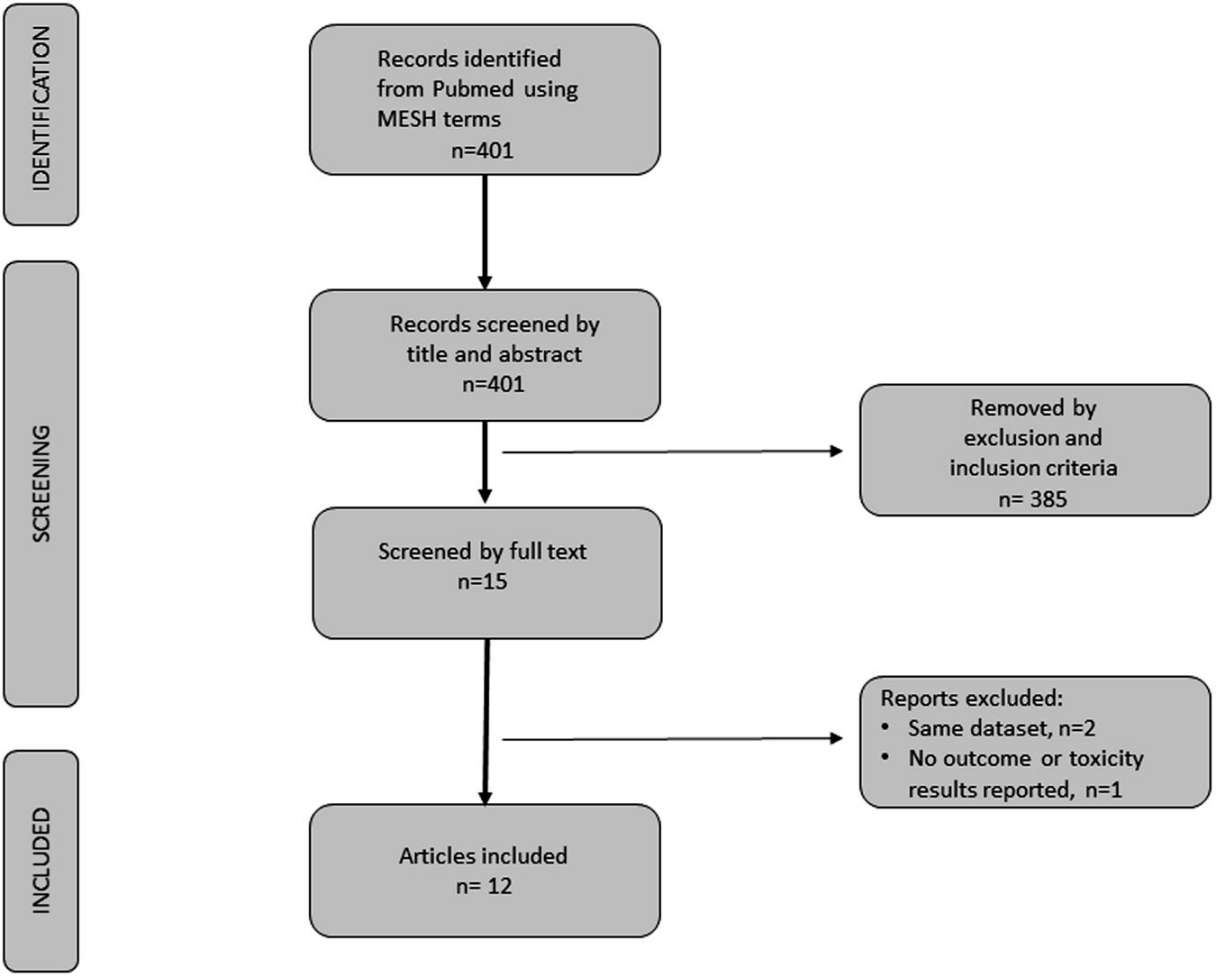
PRISMA diagram of the study selection procedure for the systematic review and meta-analysis. PRISMA: Preferred reporting items for systematic reviews and meta-analyses.

Our initial search identified 401 studies. The titles and abstracts identified via this search were screened and 15 papers were selected for full text review according to the eligibility and exclusion criteria. Of these, 3 publications were excluded (two due to redundancy in the dataset and one did not report outcomes and/or toxicity results). The study selection steps are presented in Fig. 1.

## Results

Table 1 summarizes the characteristics of the 12 publications. Most trials are phase 2 trials (*n* = 10) except for 2 being phase 3 trials [12, 13]. The primary and the salvage setting are approximately equally represented in these publications (*n* = 7 [13–19] versus *n* = 6 [12, 13, 20–23] respectively). The therapeutic combinations evaluated frequently were enzalutamide and radiotherapy (RT) (*n* = 5 [14, 15, 18, 20, 23]), and enzalutamide + ADT ± RT ± Abiraterone (*n* = 5 [12, 13, 16–18]). Finally, 3 studies had at least one arm evaluating enzalutamide only [19, 21, 22].

**Table 1:** Available prospective studies on enzalutamide in Primary and Recurrent Non-Metastatic Hormone Sensitive Prostate Cancer.

First Author Year Trial name	Phase	Setting	Study population	No pts	Median FU (mo)	Arm 1	Arm 2	Arm 3	Primary outcome(s)	Grade ≥ 3 toxicity CTCAE v4.0
Shore 2022 ENACT [19]	2	Primary	LR or IR PCa	222	24	1 y Enzalutamide + AS	AS	None	PFS 71.9% (Intervention) vs 62.8% (Control)	9.8% (Arm 1) vs 8.8% (Arm 2)
Lara 2022 ENZART [15]	2	Primary	IR PCa	62	12	6 mo Enzalutamide + RT (70 Gy/28fr)	None	None	PSA decline > 80% PSA decline in 100% of pts at the 25 w after Enzalutamide administration	Acute: 48% (during enzalutamide treatment and until 1 mo after cessation of enzalutamide) Late: 14%
Kaplan 2021 [14]	2	Primary	IR PCa	64	6	6 mo Enzalutamide + RT (75.6–79.2 Gy/42–44fr)	None	None	PSA nadir achieved during 6 mo 77% pts PSA ≤ 0.2 ng/ml, 94% pts PSA ≤ 0.5 ng/ml	19%
Shee 2022 [18]	2	Primary	HR or LA PCa	16	35.5	24 mo GnRH agonist and Enzalutamide + RT (45 Gy/25 fr prostate and pelvis + 15 Gy/1 fr prostate BT boost)	None	None	PSA complete response rate Defined as PSA nadir < 0.3 ng/mL 100% pts PSA ≤ 0.3 ng/ml	9.3%
Attard 2022 STAMPEDE [13]	Combined secondary analysis of phase 3 trials	Primary/Salvage	HR or LA PCa or relapsing with HR features NEM ( < 5% with recurrence)	1060	72	(1) 36 mo GnRH agonist/antagonist + 24 mo Abiraterone (2) 36 mo GnRH agonist/antagonist + 24 mo Abiraterone + 24 mo Enzalutamide	36 mo GnRH agonist/antagonist	None	6y-MFS 82% (Arm 1) vs 69% (Arm 2)	37% (Arm 1 (1)) 58% (Arm 1 (2)) 29–32% (Arm 2)
McKay 2019 [16]	2	Primary	IR and HR PCa	75	36	24 w Enzalutamide + leuprolide + abiraterone followed by RP	24 w Enzalutamide + leuprolide followed by RP	None	Pathological complete response rate 10% (intervention) vs 8% (control)	26% (Arm 1) vs 4% (Arm 2)
Montgomery 2017 [17]	2	Primary	IR and HR PCa	52	NA	24 w Enzalutamide + dutasteride + leuprolide followed by RP	24 w Enzalutamide followed by RP	None	Pathological complete response rate 4% (intervention) vs 0% (control)	24% (Arm 1) vs 11.1% (Arm 2)
Freedland 2023 EMBARK [44]	3	Salvage	BCR after treatment of the primary (RT or RP) NEM	1068	60.7	Enzalutamide + leuprolide	Placebo + leuprolide	Enzalutamide	5y-MFS 87.3% (Arm 1) vs 71.4% (Arm 2) vs ~80% (Arm 3)	46.5% (Arm 1) vs 42.7% (Arm 2) vs 50.0% (Arm 3)
Tran 2022 SALV-ENZA *Abstract* [23]	2	Salvage	BCR after RP NEM	86	34	6 mo Enzalutamide + RT (66.6–70.2 Gy/37–39fr)	Placebo + RT (66.6–70.2 Gy /37–39fr)	None	2y-BCRFS 84% (Arm 1) vs 66% (Arm 2)	NA
Madan 2021 [21]	2	Salvage	BCR after treatment of the primary (RT or RP) NEM	38	NA	3 mo Enzalutamide + PROSTVAC	3 mo Enzalutamide 160 mg/d	None	PSA decline in both arms > 99%	5.2%
Bitting 2021 STREAM [20]	2	Salvage	BCR after RP NEM	38	37.5	6 mo Enzalutamide + ADT + RT (66/33fr)	None	None	2y-PFS 65%	23%
Tombal 2018 [22]	2	Salvage	BCR after RP or RT with (26) or without (41) metastases	67 (42 evaluable at 3 years)	36	Enzalutamide until disease progression or unacceptable toxicity	None	None	PSA response (≥80% from baseline) 90.5% pts	34%

*AS* Active surveillance, *BCR* Biochemical relapse, *NEM* No evidence of metastases, *RP* Radical prostatectomy, *RT* Radiotherapy, *PFS* Progression-free survival, *MFS* Metastases-free survival, *BCRFS* Biochemical relapse free survival, *LR* Low risk, *HR* High risk, *LA* Locally advanced, *W* week, *Mo* month.

### Primary setting

#### As an alternative to Active Surveillance

The ENACT trial is an open-label phase 2 trial which randomized patients with low-risk or intermediate-risk PCa between active surveillance (AS) and AS plus enzalutamide monotherapy given for 1 year [19]. The primary endpoint was time to pathological or therapeutic PCa progression (pathological, ≥1 increase in primary or secondary Gleason pattern or ≥15% increased cancer-positive cores; therapeutic, earliest occurrence of primary therapy for PCa). Approximately half of the patients had low-risk disease, the other half had favorable intermediate-risk disease. With a median follow-up of 16.2 months for patients receiving enzalutamide and 8.8 months for patients undergoing AS, treatment with enzalutamide significantly reduced the risk of PCa progression by 46% vs AS (28.1% vs 37.2% respectively, HR, 0.54; 95% CI: 0.33–0.89; *P* = 0.02). During the 1-year treatment period, 92.0% of patients reported any grade toxicity in the enzalutamide group versus 54.9% in the AS group. The most frequent reported toxicity in the enzalutamide group was fatigue in more than half of the patients, gynecomastia and other-related symptoms in one third of the patients, and erectile dysfunction in 18% of patients.

When published, this study was heavily criticized, for several reasons. First, current guidelines clearly state that all active treatment options present a risk of over-treatment for the management of low-risk disease and that AS or watchful waiting (based on life expectancy) is standard of care in this setting [24]. Second, the relevance of the chosen endpoint was questioned. Giving a drug that blocks PCa growth and PSA production could delay grade progression. The fact that it could translate to benefit with stronger endpoints as metastasis-free survival (MFS) or OS is very unlikely. Based on these 2 remarks, it was deemed very questionable to provide a treatment with a high toxicity and a significant financial burden on healthcare systems without any effect on survival or quality of life, especially for patients who do not need treatment in the first place [25].

#### Neoadjuvant to surgery

Radical prostatectomy (RP) can be curative for high-risk PCa patients. However, a significant subset of high-risk PCa patients experience biochemical relapse (BCR) with an increased risk of PCa mortality [26]. In view of this, several studies have evaluated the potential benefit of adding neoadjuvant treatment such as ADT to RP. However, these studies fail to show any benefit in prostate cancer-specific survival (PCSS) or PSA relapse-free survival [27, 28], and therefore neoadjuvant ADT before RP is not recommended by the EAU guidelines [24]. Combination neoadjuvant therapy including the addition of docetaxel or ARPIs to ADT could bring benefit to high-risk PCa patients where ADT alone has failed. Two randomized phase 2 trials have investigated the role of enzalutamide alone or in combination with leuprolide and abiraterone in the neoadjuvant setting [16, 17]. In a non-comparative study, Montgomery et al. randomized 52 patients with localized PCa with Gleason Score of 7 or above or PSA > 10 ng/mL between 6 months of neoadjuvant enzalutamide in combination with dutasteride and leuprolide, and enzalutamide alone. The primary objective was to assess the pathologic complete response (pCR) rate [17]. Baseline characteristics were well balanced between the 2 treatment arms with approximately 79% of patients with a high-risk disease in each arm. In the enzalutamide alone arm, none of the 25 patients achieved pCR whereas in the enzalutamide/dutasteride/leuprolide arm, 4.3% achieved pCR. Grade ≥ 3 toxicity occurred in 11.1% of patients in the enzalutamide alone arm versus 24.0% of patients in the combination arm. Gynecomastia and mastodynia were more frequent in the enzalutamide arm than in the combination arm (63% vs. 12 and 59% vs. 8%, respectively, all grades). On the contrary, patients in the enzalutamide arm experienced fewer hot flashes than those in the combination arm (26% vs. 96%, respectively, all grades). Though non-castrating therapy alone seems ineffective in producing pathological response, combining ADT and ARPIs may represent a promising therapeutic approach. That is why, the same group conducted subsequently a randomized phase 2 trial evaluating 6 months of neoadjuvant enzalutamide and leuprolide with or without abiraterone and prednisone before RP. Patients mainly had high-risk disease (86.7%). Addition of abiraterone and prednisone increased the pCR rate by 2% (8% versus 10%, respectively), this difference being not statistically significant. No grade 4 toxicity was observed. Grade 3 toxicity was higher in the abiraterone and prednisone arm (26% vs 4%) driven by increased hypertension, and liver toxicity (increased liver enzymes). In conclusion, the benefit of combining enzalutamide with or without ADT or other ARPIs in the neoadjuvant setting prior to RP is not very clear.

#### In combination with RT

##### As an alternative to ADT in intermediate-risk PCa

Several studies showed an improved OS and PCSS with the addition of short-term ADT to RT in patients with intermediate-risk PCa compared to RT alone [29–31]. However, ADT is associated with several side effects including hot flashes, loss of libido, metabolic syndrome, and erectile dysfunction [32]. Anti-androgen monotherapy does not suppress serum testosterone production, and may thus avoid several of ADT side effects at the expense of inducing others such as painful gynecomastia. Two very similar phase 2 single arm trials have investigated PSA response and side-effects after RT and 6 months of enzalutamide in intermediate-risk PCa. Kaplan et al. included 64 patients of whom 84% had unfavorable intermediate-risk disease as defined by the National Comprehensive Cancer Network guidelines while the ENZART study conducted by Lara at al included 62 patients of whom 73.7% with unfavorable intermediate-risk disease [14, 15]. Patients in the Kaplan study received 79.2 Gy in 44 fractions, whereas those in the ENZART study received 70 Gy in 25 fractions. Defining PSA response as a PSA nadir below 0.2 ng/ml during the 6 months of enzalutamide, 79% of patients achieved a PSA response in the Kaplan trial while 100% of patients did so in the ENZART study [15]. Six months after the end of enzalutamide, 56.8% of patients continued to have PSA response (<0.2 ng/ml) in the Lara et al. study (no data is available after enzalutamide cessation in the Kaplan et al. study). Up to 48% of patients had a grade ≥3 toxicity in the Lara et al. study, mainly hypertension (33.9%) with 2 patients experiencing a grade 4 toxicity (hypertensive and liver enzyme elevation). On the contrary, no grade 4 toxicity was observed in the Kaplan et al. study and 19% of patients had a grade 3 toxicity, mainly in the form of hypertension (12%). Interestingly, toxicities associated with ADT, such as hot flashes and loss of libido, were low and predominantly grade 1 in both trials. Furthermore, Kaplan et al. documented grade 1–3 toxicity rates for erectile dysfunction at 20.3%, 12.5%, and 1.5%, respectively. Finally, there is an increase in testosterone levels compared to baseline after initiation of enzalutamide with a decrease to pretreatment level 6 months after the end of therapy.

These 2 studies showed that combining enzalutamide with RT for intermediate risk cancer is safe and effective in terms of PSA response. However, PSA response is not a surrogate for OS or PCSS and thus the real long-term impact of enzalutamide on PCa patients receiving RT remains unknown [33]. Thus, the comparison of enzalutamide with ADT in combination with RT in terms of efficacy and safety still needs to be confirmed in a randomized phase III study. The only comparison that we have so far between antiandrogen and ADT in combination with RT in terms of efficacy and side effects comes from an exploratory analysis from the CHHiP trial [34]. This non-randomized comparison showed no difference in efficacy and patient-reported outcomes according to the type of hormonal treatment, though clinician-assessed erectile function at 2 years was better in the antiandrogen group. The antiandrogen used in the CHHiP trial was bicalutamide. Since Enzalutamide is a second-generation antiandrogen and has a higher affinity to androgen receptor than bicalutamide, it might be postulated that it could be more efficacious, but this still need to be demonstrated [14].

##### In combination with ADT in high-risk and very high-risk PCa

In a meta-analysis pooling data from 2 randomized controlled phase 3 trials conducted in the Systematic Therapy in Advancing or Metastatic Prostate Cancer: Evaluation of Drug Efficacy (STAMPEDE, MRC-PR08), Attard et al. investigated the addition of abiraterone and prednisone with or without enzalutamide to ADT for nodal positive non-metastatic PCa or localized PCa with at least two high-risk features, which were defined as tumor stage T3 or T4, Gleason sum score of 8–10 and PSA concentration >40 ng/ml [13]. The first trial allocated 455 patients to the control group (ADT only) and 459 patients to combination therapy (ADT and abiraterone and prednisone), and the second trial, which added enzalutamide in the combination therapy arm, allocated 533 patients to the control group and 527 patients to combination therapy. Almost all (99%) newly diagnosed N0 and 71% of N1 patients were planned for local RT. With a median follow-up of 72 months, 6-year metastasis-free survival (MFS) improved from 69 to 82% with the addition of abiraterone and prednisone ± enzalutamide. Moreover, and though they could not rule out a small benefit from combining enzalutamide and abiraterone, the authors did not recommend this combination due to increased toxicity and costs of adding enzalutamide to abiraterone. Indeed, 37% of patients in the abiraterone trial and 57% of patients in the abiraterone and enzalutamide trial had grade ≥3 toxicity during the first 24 months. This practice changing study is responsible for the new recommendation in the EAU as well as the ASTRO ACROP guidelines to offer 24 months of abiraterone with long-term ADT and RT to cN0 cM0 patients with very high-risk features and to cN1 cM0 patients [24, 35].

The choice of an ARPI in the high-risk localized setting will likely be based on market authorization, mode of administration and convenience, side-effect profile, patient profile, drug-drug interactions, rather than based on efficacy which is expected to be pretty much the same for all these second-generation antiandrogens. The benefit of the addition of enzalutamide in the high-risk non-metastatic setting still must be formally demonstrated with a randomized phase 3 trial. The ENZARAD trial (NCT02446444) (Table 2) is currently investigating the benefit of enzalutamide in high-risk PCa treated with external beam RT (EBRT) and 2 years of ADT. Similar to abiraterone, the entry of enzalutamide in the generic drug market should broaden its use. Awaiting for these results, a small phase 2 trial investigated safety, tolerability, and efficacy (PSA complete response rate) of adding enzalutamide to ADT in high-risk localized or nodal positive non-metastatic PCa patients [18]. Definition of high-risk features (node positive or, if node negative, having at least two of the following: tumor stage T3a/b, Gleason sum score of 8–10, and PSA concentration ≥20 ng/mL, ≥33% core involvement on biopsy) was very close to the STAMPEDE criteria. Sixteen patients were enrolled, and 5 were excluded before starting treatment. Of the remaining 11 patients, all completed the 24 months follow-up and 9 of them completed the 36 months follow-up. One patient (9%) had grade 4 toxicity (seizure), and 4 patients (36.4%) had grade 3 toxicity. All patients achieved PSA complete response defined as PSA ≤ 0.3 and at 36 months, 1 out of the 9 evaluable patients presented with a biochemical recurrence as per the Phoenix criteria. This study, limited by sample size, short follow-up, and lack of control group suggests that combining enzalutamide with ADT and RT is relatively well tolerated.

**Table 2:** Ongoing clinical trials investigating enzalutamide in in Primary and Recurrent Non-Metastatic Hormone Sensitive Prostate Cancer.

Clinical trial name	Clinical trial number	Phase	Setting	Study population	Trial status	Number of participants	Arm 1	Arm 2	Primary endpoint
ENZARAD	NCT02446444	3	Primary	HR and LA PCa	Active, not recruiting	802	ADT 2 y + RT	ADT 2 y + enzalutamide 2 y + RT	MFS
NA	NCT02064582	2	Primary	HR PCa	Completed	7	ADT 6 mo + enzalutamide 6 mo + RT	None	Safety
NA	NCT02023463	1	Primary	IR and HR PCa	Active, not recruiting	25	ADT 6 mo (IR) or 24 mo (HR) + enzalutamide 6 mo + RT	None	Acute toxicities
NA	NCT05422911	2	Primary/Salvage	Patients who need ADT at HR for the development of progression of disease/metastasis	Active, recruiting	200	Abiraterone + ADT	Apalutamide + ADT or Enzalutamide + ADT	PSA progression
RTOG 3506	NCT03809000	2	Salvage	BCR after RP	Completed	242	sRT + ADT 2 y	sRT + ADT 2 y + enzalutamide 2 y	PFS
NA	NCT04409288	3	Salvage	BCR after RP or RT with or without metastases	Active, recruiting	146	12 w of apalutamide than 5 w of washed out period than 12 w of enzalutamide	12 w of enzalutamide than 5 w of washed out period than 12 w of apalutamide	Patient Preference Questionnaire
NA	NCT02278185	2	Salvage	BCR after RP or RT with or without metastases	Active, not recruiting	56	ADT 1 y	Enzalutamide 1 y	Metabolic Syndrome Incidence

*AS* Active surveillance, *BCR* Biochemical relapse, *NEM* No evidence of metastases, *RP* Radical prostatectomy, *RT* Radiotherapy, *PFS* Progression-free survival, *MFS* Metastases-free survival, *BCRFS* Biochemical relapse free survival, *LR* Low risk, *HR* High risk, *LA* Locally advanced, *W* week, *Mo* month, *Y* year.

### Salvage setting

#### In combination with RT

The addition of ADT to salvage RT as well as its duration is still an open question. Three randomized controlled trials have compared short-term ADT versus no ADT in combination with salvage RT [36–38]. The GETUG-AFU 16 was the only one showing a benefit in MFS of the addition of short-term ADT [37]. This was not seen in the RADICALS-HD [38] and RTOG 0534 [36] trials. The DADSPORT meta-analysis pooled these 3 trials to show a statistically significant but clinically irrelevant 5-year absolute improvement in MFS of 0 vs 6 months ADT of 2% (90% vs 92%, HR 0.82; 95% CI: 0.70–0.96) [39]. The only trials assessing the benefit of a long-term (2 years) regimen compared to RT alone used the first-generation antiandrogen bicalutamide 150 mg (RTOG 9601) [40, 41]. Long-term bicalutamide improved OS and MFS in patients with high baseline PSA values (>0.6 ng/ml) but was on the contrary detrimental to patients treated with early salvage RT (PSA < 0.6 ng/ml), causing an increase of other-cause mortality and late grade ≥3 toxicity hazard. Likewise, the first results of the RADICALS-HD trial suggest that in postoperative radiotherapy patients, long-term ADT improves time to salvage ADT and MFS in comparison to 6 months ADT. However, no OS benefit was shown so far [38].

In the complex context of adding ADT to salvage RT after RP, two phase 2 trials have investigated the addition of enzalutamide to salvage RT [23] or to salvage RT with ADT [20].

- In the SALV-ENZA trial, men with BCR after RP were enrolled into a randomized, phase 2, double‐blinded, placebo-controlled, multicenter study of RT plus enzalutamide or placebo, given for 6 months. The primary endpoint was freedom from PSA progression (FFPP). With a median follow-up of 34 months, FFPP was significantly improved with enzalutamide versus placebo (hazard ratio [HR], 0.42; 95% CI: 0.19 to 0.92; *P* = 0.031), and 2-year FFPP was 84% versus 66%, respectively. Apart from increased nausea and breast pain observed in the enzalutamide arm, no significant differences in grade 1 and 2 toxicity were noted between the two groups. Grade 3 toxicities were rare, with only 3 cases for the enzalutamide arm and 7 cases for the placebo arm [23].
- In the single arm phase 2 STREAM trial, patients with BCR after RP were treated with a short course of ADT and enzalutamide combined with prostate bed RT [20]. With a median follow-up of 37.5 months, they reported a 2-year PFS of 65%. Grade 3 hypertension was the most frequent grade 3 event, occurring in 11% of patients. Other grade 3 toxicities included headache, tremor, back pain, and fatigue (each 3%).

#### As Monotherapy or in combination with ADT

According to the EAU guidelines, ADT may be offered to patients as monotherapy in selected cases with BCR [24]. Intensification of ADT with enzalutamide or replacement of ADT by enzalutamide are 2 pathways that are currently investigated in this setting:

- Tombal et al. were the first to investigate enzalutamide monotherapy as the front-line treatment in patients with localized PCa and mCSPC [22, 42, 43]. In a phase 2 multicenter open label single arm study, 67 patients (35 M0, 10 M1, and 22 Mx) received enzalutamide monotherapy until disease progression or unacceptable toxicity development. To assess the efficacy of enzalutamide, they used PSA response defined as 80% PSA drop over pretreatment values. Of the 42 patients who remained on enzalutamide up to the 3-year visit, 38 (90.5%) maintained a PSA response. At 3 years, there was a small decrease in bone mineral density (BMD) compared to baseline, lower than the mean BMD decrease reported for long-term ADT. Nine out of 67 patients (13.4%) had adverse events possibly related to enzalutamide which led to enzalutamide discontinuation during or after 3 years. Gynecomastia and fatigue were the 2 most frequent adverse events (49.3% and 38.8% respectively, all grades). During the 3 years, grade ≥3 toxicity was reported in 34% of patients. The open label single arm design and the small and heterogenous study population are among the limitations of this study. However, it provided rationale for further investigation of enzalutamide monotherapy in the nmCSPC setting, which was subsequently carried out with the EMBARK trial.
- The EMBARK trial included 1068 patients with high-risk BCR (PSA doubling time (PSADT) ≤ 9 months) after local treatment and no evidence of distant metastases on conventional imaging. Patients were randomized between ADT, ADT plus enzalutamide, and enzalutamide alone [12, 44]. In this trial, after a median follow-up of 60.7 months, enzalutamide + ADT demonstrated a statistically significant improvement in 5-year MFS versus ADT + placebo (87.3% vs 71.4%, HR, 0.42; 95% CI: 0.31–0.61; *P* < 0.0001). Interestingly, enzalutamide monotherapy also significantly improves MFS versus ADT (HR 0.63; 95% CI: 0.46−0.87; *P* = 0.0049). Of all patients, 164 (46.5%) patients experienced a grade ≥3 adverse events in the combination arm, versus 151 (42.7%) in the ADT + placebo arm, and 177 (50.0%) in the enzalutamide alone arm. More patients experienced gynecomastia in the enzalutamide monotherapy arm than in the ADT ± enzalutamide arms. In conclusion, enzalutamide, either alone or in combination with ADT, may be beneficial for high-risk BCR patients without metastasis. However, it’s important to note that the Embark study only utilized conventional imaging, leaving the effectiveness of PSMA PET/CT unaddressed, despite its emerging role in detecting prostate cancer recurrence, even in low levels of PSA [45].

## Discussion

Enzalutamide was the first second-generation ARTA to receive FDA approval and is now approved in combination with ADT for the treatment of castration-resistant PCa, regardless of the presence of metastases, and as first-line therapy in mCSPC [5, 6, 8–10]. A growing number of studies have examined the effectiveness and safety of enzalutamide when used in earlier stage of the disease. In this systematic review, we summarize the use of enzalutamide in non-metastatic hormone sensitive prostate cancer.

Conventionally, enzalutamide is used in addition to ADT, in populations where ADT alone or in combination with RT would be the SOC. The idea is to reinforce the systemic treatment, a strategy which has been conducted in other settings resulting in better oncological outcomes [5, 6, 8–10]. In the primary setting, this idea is particularly relevant for high-risk and very high-risk PCa patients treated with a combination of long-term ADT and RT. Attard et al. changed the SOC treatment for this group of patient with the results of the STAMPEDE trial [13] and now the addition of abiraterone to ADT is recommended for N0 patients with high risk features or N1 patients [24]. We expect to have the same benefit demonstrated in the ENZARAD trial (NCT02446444) with the addition of enzalutamide to long-term ADT and RT for this group of patients.

In the same primary setting, combining ADT and enzalutamide before RP is being explored in some studies [16, 17]. However, this approach remains investigational and is very unlikely to be practice changing. In the salvage setting, the benefit of adding enzalutamide on top of SOC treatment needs to be further investigated. For patients relapsing after RP treated at a low PSA level (<0.5 ng/ml) with salvage prostate bed RT, it is already very debatable to add ADT. Indeed, the DADSPORT meta-analysis specifically investigated the impact of the addition of ADT to salvage RT in terms of OS and showed no evidence of an OS benefit with ADT *vs* none, irrespective of whether 6 months or 24 months of ADT but only a modest MFS benefit on the long-term FU (2% with short-term ADT at 5-yr and 6% with long-term ADT at 10-y) [39]. This meta-analysis also underlines the good prognosis of PCa patients treated with salvage RT alone with MFS rates exceeding 70% at 10 years. In a situation when adding ADT does not bring any survival benefit, a combination of ADT and enzalutamide is therefore highly questionable. However, for patients with a high PSA level at relapse and a short PSADT (≤9 months), the benefit of intensifying ADT with enzalutamide compared to ADT alone has been proven in the EMBARK trial with an increased MFS in the combination arm compared to the leuprolide arm. These patients did not have any metastases based on conventional imaging. However, with the advance of molecular imaging, it is expected a high proportion of these patients would exhibit metastatic or pelvic lesions on PSMA PET/CT and may thus benefit from radiotherapy [45].

Though the efficacy of enzalutamide seems to be equal if not superior to ADT in the EMBARK trial [12], the toxicity may not be decreased. It is indeed very tempting to believe that because enzalutamide does not lower testosterone level, it is better tolerated than ADT. However, in the EMBARK trial, grade ≥3 adverse events were present in 50.0% of patients in the enzalutamide monotherapy arm versus 42.7% in the leuprolide arm. If we investigate the type of adverse events by treatment arm, we observe more frequent hot flashes in the leuprolide arm than in the enzalutamide arm (57.3% vs 21.8%). On the contrary, fatigue was higher in the enzalutamide arm than in the leuprolide arm (46.6% vs 32.8%), as well as gynecomastia (44.9% vs 9.0%), and ischemic heart disease (9.0% vs 5.6%). Despite enzalutamide being more effective than ADT in the EMBARK trial, the use of enzalutamide in place of ADT in populations where ADT use is the standard of care needs to be further investigated.

## Conclusions

In summary, our results highlight the different therapeutic strategies trying to involve enzalutamide at an earlier stage of Pca treatment. In the nmCSPC setting, using enzalutamide instead of ADT or in combination with ADT to intensify systemic treatment are 2 options that are currently investigated. For the first option using enzalutamide instead of ADT, the equivalence or superior efficacy of enzalutamide compared to ADT still needs to be demonstrated. Moreover, the toxicity of enzalutamide may not be lower than that of ADT but just different. For the second option, adding enzalutamide to ADT in order to intensify the systemic treatment should be reserved for very high-risk PCa patients, as per the STAMPEDE population [13], or relapsing disease following primary treatment with high-risk features including high PSA level at relapse and short PSADT [12]. ARPIs have drastically changed the treatment landscape of mCSPC and both metastatic and nmCRPC. Their incorporation earlier on in the disease stage was just a matter of time and STAMPEDE [13] and EMBARK [12] trials are for now the most successful examples in this regard. However, one must be very careful about the risk of overtreatment given the unnecessary toxicity it brings to patients. In the metastatic setting, de-intensification is currently being studied, notably with the EORTC-2238 GUCG (De-ESCALATE) NCT05974774, revisiting the concept of intermittent ADT in patients achieving a good PSA response after 6–12 months of ADT and one of the ARPIs. To avoid de-intensification trials in the non-metastatic setting, indications for treatment intensification should be carefully thought. Several studies are ongoing or have been recently completed further investigating the efficacy and safety of Enzalutamide in the definitive or salvage setting (Table 2). The results of these studies will further inform clinical practice in the next decade.
